# Unit-specific calibration of Actigraph accelerometers in a mechanical setup – Is it worth the effort? The effect on random output variation caused by technical inter-instrument variability in the laboratory and in the field

**DOI:** 10.1186/1471-2288-8-19

**Published:** 2008-04-11

**Authors:** Niels C Moeller, Lars Korsholm, Peter L Kristensen, Lars B Andersen, Niels Wedderkopp, Karsten Froberg

**Affiliations:** 1 Institute of Sport Science and Clinical Biomechanics, University of Southern Denmark, Odense, Denmark; 2 Department of Statistics, University of Southern Denmark, Odense, Denmark; 3 The Back Research Center, Ringe, Denmark; 4 Department of Sports Medicine, Norwegian School of Sport Sciences, Oslo, Norway

## Abstract

**Background:**

Potentially, unit-specific in-vitro calibration of accelerometers could increase field data quality and study power. However, reduced inter-unit variability would only be important if random instrument variability contributes considerably to the total variation in field data. Therefore, the primary aim of this study was to calculate and apply unit-specific calibration factors in multiple accelerometers in order to examine the impact on random output variation caused by inter-instrument variability.

**Methods:**

Instrument-specific calibration factors were estimated in 25 MTI- and 53 CSA accelerometers in a mechanical setup using four different settings varying in frequencies and/or amplitudes. Calibration effect was analysed by comparing raw and calibrated data after applying unit-specific calibration factors to data obtained during quality checks in a mechanical setup and to data collected during free living conditions.

**Results:**

Calibration reduced inter-instrument variability considerably in the mechanical setup, both in the MTI instruments (raw SD_between units _= 195 counts*min^-1 ^vs. calibrated SD_between units _= 65 counts*min^-1^) and in the CSA instruments (raw SD_between units _= 343 counts*min^-1 ^vs. calibrated SD_between units _= 67 counts*min^-1^). However, the effect of applying the derived calibration to children's and adolescents' free living physical activity data did not alter the coefficient of variation (CV) (children: CV_raw _= 30.2% vs. CV_calibrated _= 30.4%, adolescents: CV_raw _= 36.3% vs. CV_calibrated _= 35.7%). High correlations (r = 0.99 & r = 0.98, respectively) were observed between raw and calibrated field data, and the proportion of the total variation caused by the MTI- and CSA monitor was estimated to be only 1.1% and 4.2%, respectively. Compared to the CSA instruments, a significantly increased (9.95%) mean acceleration response was observed post hoc in the batch of MTI instruments, in which a significantly reduced inter-instrumental reliability was observed over time.

**Conclusion:**

The application of unit-specific calibration factors to data collected during free living conditions had no apparent effect on inter-instrument variability. In all probability, the effect of technical calibration was primarily attenuated in the field by other more dominant sources of variation. However, routine technical assessments are still very important for determining the acceleration responses in the batch of instruments being used and, if performed after every field use, for preventing decidedly broken instruments from being returned into the field repeatedly.

## Background

A valid, reliable, and feasible assessment technique is essential when trying to provide information about important aspects of physical activity (PA) in children and young people (e.g. describe trends in the level of PA, examine tracking of PA, establish indications of early links between PA and health status, etc.). Over the years children's and young people's levels of PA have typically been assessed by interviews [1,2], heart rate monitoring [3,4], and in particularly by the use of questionnaires [5-7]. However, quantifying PA by the use of subjective statements are influenced, and limited by, cognitive differences among the participants under study, and self-report measures are considered inappropriate in particular when applied to children [8]. Due to the lack of reliability of self-report measures, and the emotional and fitness caused bias of heart rate monitoring, objectively registration of PA with motion sensors is generally regarded as an improved measurement technique for monitoring PA in large scale population studies, especially when children are the target group.

Accelerometers provide objective information on PA duration, frequency, and intensity, and are being increasingly used to monitor levels of PA [9-11]. Therefore, quantifying instrument validity and reliability has become an issue of growing interest and importance.

Raw accelerometer output is usually measured in a proprietary and arbitrary unit called accelerometer counts. As such, most users convert counts to a more meaningful indicator of PA. Therefore, several studies have been initiated in order to validate how these accelerometer counts are related to different types of activities and/or different intensity thresholds [12-17], as well as to overall or activity specific energy expenditure [18-22].

Most manufacturing companies perform a calibration check before shipping in an order to ensure that different units provide a similar response to a standardized acceleration. However, this type of technical calibration has rarely been described by the manufactures in sufficient details, and as a consequence some research groups began to conduct their own calibration [23]. On the other hand, most research teams do not incorporate unit-specific calibration into their study protocol, and instead extensive calibration is often performed only when broken instruments are returned to the manufactures for repair.

Only few studies have been conducted in which technical reliability has not only been assessed but also separated from biological variability [24-26]. However, unit-specific calibration has been shown to be necessary in order to minimize the inter-instrument output differences observed under standardized conditions in mechanical setups [27,28]. However, reducing inter-instrument variability through technical/mechanical calibration would only be important in order to improve field data quality and study power if random variability across units contributed considerably to the total variation in field data. Otherwise, the primary focus can shift to other sources of variation (e.g. variation over time, or position worn on the body including compliance with the instructions given how to wear the accelerometer).

Therefore, the primary aim of the present study was to calculate and apply unit-specific calibration factors in multiple accelerometers units from different batches of purchase in order to examine the impact of calibration on random output variation in controlled laboratory conditions and in the field, respectively.

Furthermore, post hoc analyses were conducted in order to examine possible inter-instrumental changes over time, and whether the acceleration response differed across different generations of instruments.

## Methods

### Instrumentation

Mechanical movement and free living habitual physical activity (HPA) was assessed with the Actigraph Model 7164 accelerometer (Actigraph LLC, Pensacola, FL). The Actigraph was originally called the Computer Science Applications (CSA) accelerometer as it was named after the company that manufactured it. However, the CSA changed names to the MTI after the technology was purchased by Manufacturing Technologies Inc. The Actigraph monitor is a uniaxial piezo-electric accelerometer designed to measure and record accelerations along the vertical axis of the body ranging in magnitude from 0.05 to 2.13 g. Instrument specifications have been described in more detail by Tryon & Williams [29].

The inter-instrument reliability in a total number of 78 accelerometers was examined separately in two subgroups consisting of 25 instruments purchased new in 2003 (from hereon referred to as MTI) and 53 instruments purchased new in 1997 (from hereon referred to as CSA).

### Experimental Laboratory calibration

Inter-instrument reliability was examined under standardized conditions in a mechanical setup in the laboratory before and during the data collection period in the Danish part of European Youth Heart Study II [30]. Subsequently, individual calibration factors were derived for all units as instrument outputs were checked and compared.

#### Mechanical laboratory setup

The calibration machine used in the experimental setup in the laboratory consists of two rotating wheels, both rotating with the same constant angular velocity (ω). The wheels are connected by a rod (CR) and driven by an electric motor. The accelerometer units are attached to a plate on the rod during the calibration procedure. Attachments of the rod is placed away from the centre of the rotating wheels meaning that the instruments will experience accelerations and decelerations with a vertical displacement equal to two times the length of the radius (*r*) from the centre to the point of attachment. ω (radians/sec) is directly related to the movement frequency *f *in Hertz by the equation: ω = 2*π*f

The radius, or two times the length of movement, is restricted to three different settings (22.0, 35.5, and 49.0 mm.) in this mechanical setup, which together with the fully adjustable movement frequency will regulate acceleration values according to the following equation: A(t) = 8*r*π*f^2^

**Figure 1:**
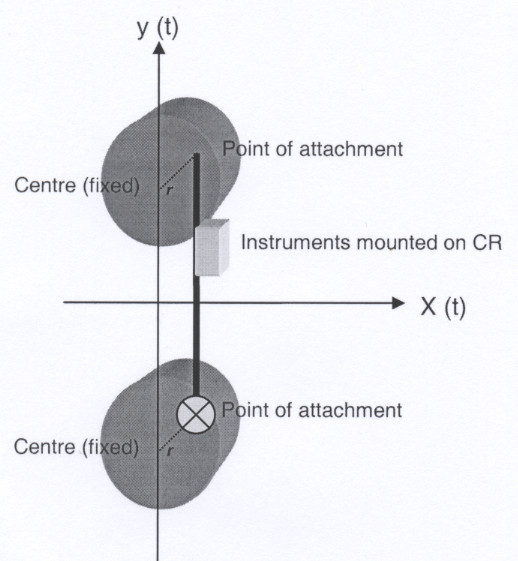
Calibration machine used in the laboratory with abscissa, X(t) and ordinate, Y(t). CR is the vertical connecting rod and *r *is the radius.

The mechanical setup, which preciously has been described and used by Brage et al. [27], is illustrated in Figure 1.

#### Full laboratory calibration protocol

All units were calibrated in four different settings varying in frequencies and/or amplitudes, which on average produced accelerometer outputs roughly equal to 3000, 5000, 3000, and 8000 counts*min^-1^, respectively. These settings were established to produce a range of physiologically relevant accelerometer count outputs frequently observed during free-living activities. Calibration was performed at three different time points (i.e. November 2003, January 2004, and March 2004). When performing calibrations in the mechanical setup the epoch was set at 60 s, which comprised an integral of 600 measurements. In order to ensure full epochs output values for each MTI/CSA, units were expressed as the mean counts*min^-1^of minutes 2–8 in each trial, which in total lasted 10 minutes.

Initially, ten instruments were randomly selected to identify appropriate frequencies and amplitudes producing accelerometer outputs of 3000, 5000, 3000, and 8000 counts*min^-1^, respectively. The mean of the frequencies derived in the ten different units at the different amplitudes, where the desired instrument outputs were produced, were used as the "gold standard" frequencies for the whole population of instruments.

Since group-differences had been observed between MTI- and CSA instruments when acceleration responses were checked initially before the field data collection period just to make sure that all units were functional, "gold standard" frequencies were calculated and used separately for the two groups of instruments. Subsequently, unit-specific calibration was performed in all units as acceleration responses were analysed using the "gold standard" frequencies. The unit-specific calibration factors were estimated by dividing the mean acceleration response within the whole population of instruments within a group by the individual instrument acceleration responses. A mean of the unit-specific calibration factors derived in the four different settings at the three different time points was used as the final unit-specific calibration factor used for further analyses in this study.

In order to reduce the random variation in the experimental setup in the laboratory, frequencies were adjusted within 0.01 Hz, and each individual instrument was allocated one specific position of attachment to the plate at the calibration machine, which was retained during the entire study.

The different calibration settings used for the MTI/CSA instruments in the mechanical setup are shown in Table 1.

**Table 1:** Four different calibration settings for the MTI/CSA, varying in Frequency, radius, and acceleration

**MTI instruments**				
**Setting**	**Frequency (Hz)**	**Radius (mm)**	Acceleration (m*s^-2^)	Output (counts*min^-1^)

#1	1.657	22.0	1.52	≈ 3000
#2	1.537	35.5	2.08	≈ 5000
#3	0.950	49.0	1.11	≈ 3000
#4	1.657	49.0	3.38	≈ 8000

**CSA instruments**				

#1	1.705	22.0	1.61	≈ 3000
#2	1.578	35.5	2.22	≈ 5000
#3	0.970	49.0	1.16	≈ 3000
#4	1.717	49.0	3.63	≈ 8000

### Field study design

#### Participants

Field data was collected in 458 third grade children (259 girls and 199 boys) aged 8–10 years and in 444 ninth grade adolescents (251 girls and 193 boys) aged 14–16 years. The children and adolescents were randomly sampled from schools stratified according to location and the socio-economic character of its uptake area. A more thorough description of the sampling procedure used in The European Youth Heart Study has been described elsewhere [30].

#### Measurement protocol and data reduction

Twenty five different MTI units were distributed to a total of 425 third grade children, and 53 different CSA units were distributed to a total of 444 ninth grade adolescents, when the HPA level was monitored during the academic year in 2003/04. Children/adolescents were asked to wear the accelerometer for at least five consecutive days, including at least one weekend day. The accelerometers were returned by the children/adolescents and data downloaded on the day of their physical examination.

The data reduction program, which was set up to analyse activity data on a daily basis, revealed significantly different HPA levels between weekdays and weekend days. Therefore, children's and adolescent's HPA were weighted according to day types. In children, "activity" between 00.00 and 06.00 h was cut away in all data files in order to avoid biased data, caused by the fact that some children forgot to take off the accelerometer during sleep. Some adolescents stayed up late at night, especially in the weekends, and therefore, all data files recorded in ninth grade adolescents were checked manually in order to decide whether activity between 00.00 and 06.00 h should be removed or not. HPA data were included for further analyses if the person had accumulated a minimum of 10 hours of activity data per day, for at least 3 days, including both weekdays and weekend days. A more detailed description of the day type adjustments and the manual check of data files have been described elsewhere [31].

### Effect of applying calibration factors

Unit-specific calibration factors were applied to a) data derived in the mechanical setup in the laboratory as all units were checked in setting #2 each time they were returned from the field during the field data collecting period in order to ensure that the instruments maintained properly function, and to b) data collected during free-living conditions in the field.

The effect of calibration on random variation caused by inter-instrument variability was analysed by comparing characteristics of raw and calibrated data.

### Post hoc examination based on observations in the laboratory

Inspired by the observations observed during calibration in the laboratory, post hoc analyses were applied in order to analyse if a) inter-instrument reliability differed between the groups of MTI- and CSA instruments, and if b) inter-instrument reliability changed over time. Furthermore, it was analysed whether acceleration responses (acceleration/accelerometer output) differed systematically between the groups of MTI- and CSA instruments as the accelerations needed to produce one thousand accelerometer counts per minute were estimated for all units in all four settings at the three different time points in the mechanical setup.

### Statistics

One-way analysis of variance estimating the standard deviation (SD) between and within instruments was used to describe the potential for increasing inter-instrument reliability through unit-specific calibration, and to demonstrate the effect of calibration on random output variation caused by inter-instrument variability in the mechanical setup in the laboratory. Furthermore, the effect of calibration was examined by comparing the SD and the coefficient of variation (CV) of the raw and calibrated accelerometer output derived in the mechanical setup and in the field during free-living conditions, respectively.

The amount of variation introduced to or removed from field data when applying unit-specific calibration factors was estimated by dividing the variance of the delta instrument output between raw and calibrated field data by the total variation in field data. Pearson's correlation coefficients were calculated in order to describe the association between raw and calibrated accelerometer output in the field, and a Bland-Altman plot was additionally applied in order to assess the individual agreement between raw and calibrated field data.

The homogeneity of the SD on the output was tested Post hoc using a likelihood ratio test estimated by a proc mixed command in SAS (version 9.0), in order to analyse if inter-instrument reliability changed over time. The same approach was used to verify whether inter-instrument reliability differed between MTI and CSA instruments. Furthermore, multiple regressions with robust standard errors were used to test if acceleration responses differed between MTI and CSA instruments, when adjusting for calibration setting and time of calibration.

All statistical analyses, except the likelihood ratio tests, were performed using STATA 8.0.

### Ethics

All parents gave written informed content for their child to participate, and all children/adolescents gave verbal consent. The study was approved by the local scientific ethics committee and follows the rules and principles stipulated by the Helsinki declaration.

## Results

### Potential for increasing inter-instrumental reliability in the laboratory

**Figure 2:**
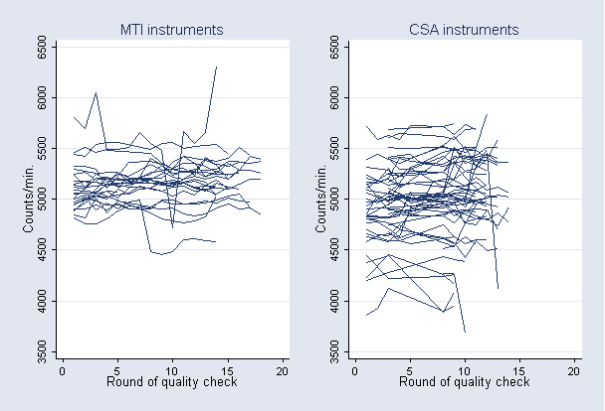
Raw instrument output by round of quality check. Within MTI units variation: SD = 112 counts*min^-1^. Between MTI units variation: SD = 195 counts*min^-1^. Within CSA units variation: SD = 120 counts*min^-1^. Between CSA units variation: SD = 343 counts*min^-1^.

Examining the effect of unit-specific calibration on the random variation in HPA would be relevant only if the calibration performed is expected to improve inter-instrument reliability. The potential for increasing inter-instrumental reliability through unit-specific calibration is depicted in Figure 2, where the raw unit-specific accelerometer output is plotted against the different rounds of quality checks performed in setting #2 in the mechanical setup.

Substantial variation between the different units, but also a considerable fluctuation within each single instrument (intra-instrument variation), can be observed over time in Figure 2. However, the SD representing the variation between units was about 2 and 3 times the size of the SD representing the variation within MTI and CSA instruments, respectively. This indicates some potential for increasing inter-instrumental reliability through the performance of unit-specific calibration in the laboratory.

### Effect of applying unit-specific calibration factors to data derived during quality checks in the mechanical setup in the laboratory

The impact on between unit variation observed in the laboratory after applying unit-specific calibration factors to data obtained in setting #2 in the mechanical setup can be seen in Table 2. Across the population of MTI instruments, the SD was reduced considerably from 221 (95% CI 206–239) counts*min^-1 ^to 127 (95% CI 118–138) counts*min^-1^, and the CV was reduced from 4.3% to 2.5%. Across the population of CSA instruments, the SD was reduced dramatically from 361 (95% CI 338–388) counts*min^-1 ^to 137 (95% CI 128–137) counts*min^-1^, and the CV was reduced from 7.2% to 2.8% after applying unit-specific calibration factors to the accelerometer output from each unit.

**Table 2:** Raw and calibrated instrument output derived through repeated measurements in setting #2 in the mechanical setup. Data are Means and standard deviations with 95% CI, and coefficients of variation

	**N**	Measured acceleration (counts*min^-1^**)**	**SD**	**CV (%)**
**Raw MTI output**	340	5160 (5137–5184)	221 (206–239)	4.3
**Calibrated MTI**	340	5155 (5142–5169)	127 (118–138)	2.5
**Raw CSA output**	407	5025 (4990–5061)	361 (338–388)	7.2
**Calibrated CSA output**	407	4927 (4913–4940)	137 (128–147)	2.8

(N equals the total number of repeated quality checks in setting #2)

**Figure 3:**
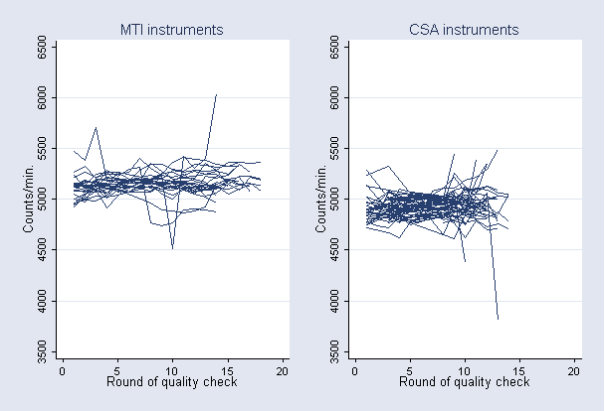
Calibrated instrument output by round of quality check. Within MTI units variation: SD = 110 counts*min^-1^. Between MTI units variation: SD = 65 counts*min^-1^. Within CSA units variation: SD = 119 counts*min^-1^. Between CSA units variation: SD = 67 counts*min^-1^.

When examining the impact of calibration on between units variation in the laboratory by comparing the SD of the instrument effect and within the instruments separately, results showed that the SD representing the variation between units in the group of MTI- and CSA instruments was approximately one half of the SD representing the variation within units after calibration (Figure 3). Therefore, based on information observed in the mechanical setup it seems reasonable to perform a technical unit-specific experimental calibration in the laboratory. On the other hand, considerable inter-instrument variability still exists across the two batches of instruments (p < 0.0001), even after performing unit-specific calibration.

### Valid activity files collected during free living conditions in the field

Valid HPA data, obtained with MTI accelerometers, was obtained in 389 third grade children (227 girls and 162 boys), and 296 ninth grade adolescents (172 girls and 124 boys) had valid data recorded by CSA accelerometers.

### Effect of applying unit-specific calibration factors to field data

The effect of calibration on random variation caused by inter-instrument variability in field data can be seen in Table 3. In third grade children measured with MTI instruments, the SD and CV remained unchanged after calibration (SD_raw _= 219 (95% CI = 205–236) counts*min^-1 ^vs. SD_calibrated _= 220 (95% CI = 206–237) counts*min^-1^, CV_raw _= 30.2% vs. CV_calibrated _= 30.4%). The amount of variation introduced by applying unit-specific calibration factors to HPA data was estimated to account for only 1.1% of the total variation in children's field data.

**Table 3:** Raw and calibrated instrument output recorded during free-living conditions in the field. MTI readings are obtained in third grade children and CSA readings are obtained in ninth grade adolescent. Data are means and standard deviations with 95% CI, and coefficients of variation.

	**N**	Measured acceleration (Counts*min^-1^**)**	**SD**	**CV (%)**
**Raw MTI output**	389	724 (702–745)	219 (205–236)	30.2
**Calibrated MTI output**	389	724 (702–746)	220 (206–237)	30.4
**Raw CSA output**	296	446 (428–465)	162 (150–176)	36.3
**Calibrated CSA output**	296	440 (422–458)	157 (146–171)	35.7

(N equals numbers of children/adolescents)

In ninth grade adolescents measured with CSA instruments, the SD decreased marginally from 162 (95% CI = 150–176) counts*min^-1 ^to 157 (95% CI = 146–171) counts*min^-1 ^after calibration. Raw CV decreased by a minimum from 36.3% to 35.7% when calibrated. Additionally, the impact of calibration was estimated to be 4.2% when compared to the total amount of variation observed in adolescent's field data.

**Figure 4:**
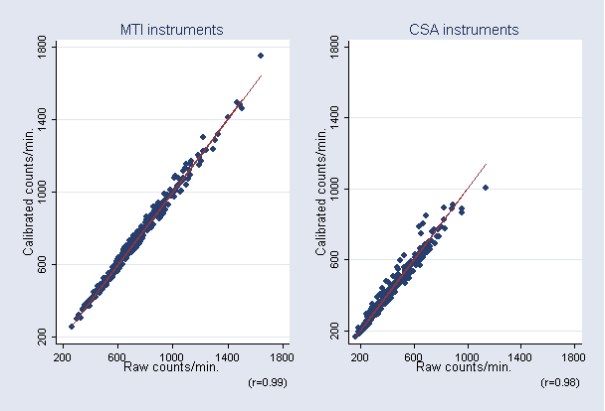
Raw instrument output plotted against calibrated instrument output. Data are obtained in the field.

**Figure 5:**
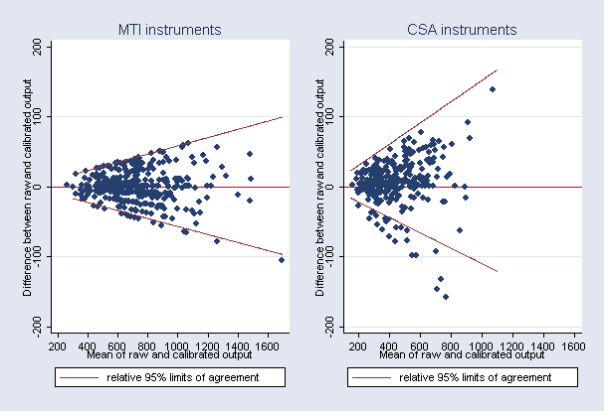
Limits of agreement (Bland-Altman plot) between raw and calibrated instrument output. Data are obtained in the field.

Correlations, including the line of equality, and the relative 95% limits of agreement between raw and calibrated accelerometer output obtained in the field can be seen in Figure 4 &5, respectively. Calibrated accelerometer output was found to be highly correlated to raw accelerometer output in both third grade children and in ninth grade adolescents (r = 0.99 & r = 0.98, respectively), and the relative 95% limits of agreement were approximately ± 5.5% and ± 13% in children and adolescents, respectively.

### Post hoc examinations performed on the basic of observations when performing calibration

#### Instrument responses when exposed to "gold standard" frequencies in the laboratory

Table 4 and Table 5 show the mean accelerometer outputs obtained in the MTI and CSA units when exposed to gold standard frequencies during calibration in the mechanical setup. Non-homogeneous standard deviations (p = 0.00001) in the group of MTI instruments, as denoted by increasing values over time during November, January, and March, indicates a reduced inter-instrument reliability over time for all settings jointly (Table 4). In the group of CSA instruments, no significant heterogeneity was observed (p = 0.95), although the SD increased slightly during the period (Table 5).

**Table 4:** MTI instrument output derived when exposed to "golden standard" accelerations in the mechanical setup. Data are means and standard deviations with 95% CI, and coefficients of variation

**November 2003**	**N**	Measured acceleration (Counts*min^-1^**)**	**SD**	**CV (%)**
Setting #1 (1.52 m*s^-2^)	25	3023 (2991–3054)	76 (59–106)	2.5
Setting #2 (2.08 m*s^-2^)	25	5015 (4960–5071)	134 (105–187)	2.7
Setting #3 (1.11 m*s^-2^)	25	2986 (2958–3013)	67 (53–94)	2.2
Setting #4 (3.38 m*s^-2^)	25	7967 (7876–8058)	219 (171–305)	2.7

**January 2004**				

Setting #1 (1.52 m*s^-2^)	25	3083 (3029–3137)	132 (103–183)	4.2
Setting #2 (2.08 m*s^-2^)	25	5133 (5046–5219)	210 (164–292)	4.1
Setting #3 (1.11 m*s^-2^)	25	3070 (3012–3128)	141 (110–196)	4.6
Setting #4 (3.38 m*s^-2^)	25	8175 (8035–8315)	339 (265–472)	4.1

**March 2004**				

Setting #1 (1.52 m*s^-2^)	23	3100 (3034–3167)	153 (119–217)	5.0
Setting #2 (2.08 m*s^-2^)	23	5142 (5042–5241)	230 (178–325)	4.5
Setting #3 (1.11 m*s^-2^)	23	3048 (2988–3108)	139 (108–197)	4.6
Setting #4 (3.38 m*s^-2^)	23	8160 (8013–8308)	341 (264–483)	4.2

(N equals numbers of instruments)

**Table 5:** CSA instrument output derived when exposed to "gold standard" accelerations in the mechanical setup. Data are means and standard deviations with 95% CI, and coefficients of variation

**November 2003**	**N**	Measured acceleration (Counts*min^-1^**)**	**SD**	**CV (%)**
Setting #1 (1.61 m*s^-2^)	50	2952 (2883–3021)	243 (203–303)	8.2
Setting #2 (2.22 m*s^-2^)	50	4861 (4757–4965)	366 (306–456)	7.5
Setting #3 (1.16 m*s^-2^)	50	2952 (2887–3017)	229 (191–285)	7.8
Setting #4 (3.63 m*s^-2^)	50	7831 (7672–7990)	560 (468–698)	7.2

**January 2004**				

Setting #1 (1.61 m*s^-2^)	53	2970 (2900–3040)	253 (212–312)	8.5
Setting #2 (2.22 m*s^-2^)	53	4883 (4775–4992)	393 (330–486)	8.0
Setting #3 (1.16 m*s^-2^)	53	2981 (2916–3045)	234 (197–290)	7.8
Setting #4 (3.63 m*s^-2^)	53	7857 (7689–8026)	611 (512–756)	7.8

**March 2004**				

Setting #1 (1.61 m*s^-2^)	53	3021 (2945–3097)	275 (231–341)	9.1
Setting #2 (2.22 m*s^-2^)	53	4966 (4852–5080)	414 (347–512)	8.3
Setting #3 (1.16 m*s^-2^)	53	3014 (2944–3084)	253 (212–313)	8.4
Setting #4 (3.63 m*s^-2^)	53	7967 (7796–8138)	620 (521–768)	7.8

(N equals numbers of instruments)

The MTI units displayed an improved level of inter-instrument reliability in comparison with the CSA units (p < 0.0001) when comparing the SD.

Finally, the MTI instruments on average needed significantly less (9.95%) acceleration to produce one thousand counts per minute when comparing the acceleration responses in the group of MTI and CSA units across all settings and time points (Table 6).

**Table 6:** Accelerations (m*s^-2^) needed to produce 1000 accelerometer count*min^-1^. Data are means and standard deviations calculated in four different settings at three different time points in the mechanical setting.

	**Setting #1**	**Setting #2**	**Setting #3**	**Setting #4**
**MTI (November)**	0.5026 (0.0124)	0.4144 (0.0110)	0.3724 (0.0084)	0.4245 (0.0117)
**CSA (November)**	0.5483 (0.0476)	0.4599 (0.0365)	0.3950 (0.0329)	0.4658 (0.0345)
**MTI (January)**	0.4932 (0.0199)	0.4053 (0.0157)	0.3627 (0.0157)	0.4141 (0.0162)
**CSA (January)**	0.5453 (0.0492)	0.4582 (0.0388)	0.3913 (0.0332)	0.4647 (0.0374)
**MTI (March)**	0.4908 (0.0245)	0.4048 (0.0187)	0.3654 (0.0172)	0.4149 (0.0175)
**CSA (March)**	0.5368 (0.0534)	0.4509 (0.0408)	0.3874 (0.0360)	0.4583 (0.0374)

When compared to the CSA units the MTI instruments, on average, needed significantly (p < 0.001) less acceleration in order to produce 1000 counts*min^-1^

## Discussion

To our knowledge, no other study has examined and compared the effect of calibration on inter-instrument reliability after applying unit-specific calibration factors to data obtained both in the laboratory and in the field.

As the primarily finding, this study revealed that unit-specific calibration factors shown to reduce inter-instrumental variability considerably in the experimental setup in the laboratory should be considered as rather ineffectual when applied to field data in children and adolescents. Furthermore, a significantly reduced inter-instrument reliability was observed over time post hoc in the MTI monitors, and when compared to the CSA instruments a significantly increased (9.95%) mean acceleration response was observed in the batch of MTI instruments. These findings should be interpreted in the light of several considerations.

### General strengths and limitations

The strengths in the present study include the large number of accelerometers examined in a mechanical setup producing highly standardized reference acceleration values. On the other hand, serving as a limitation the calibration machine in the laboratory solely offers an isolated and standardized sinusoidal way of movement, which potentially will affect the comparability of inter-instrument variability estimated according to mechanical movements in the laboratory and inter-instrument variability experienced in the field when exposed to complex human locomotion. Therefore, in order to improve the variation and complexity of movement in the mechanical setup all instruments were calibrated in three different radius settings using four different frequencies, which produced a total of four different acceleration values.

Unit-specific acceleration response varies over time (intra-instrument variation), although intra-instrument reliability has been reported to be fairly good at any given time point [26,27]. Therefore, the calibration factors estimated in this study will include residual unit-specific test-retest variation. In this study, unit-specific acceleration responses were assessed at three different time points during the period November 2003 to March 2004 in order to minimize the effect of intra-instrumental variation. Examining acceleration responses within a rage of different accelerations in multiple units more frequently than performed in the present study becomes rather problematic, if calibration should be feasible in large scale population studies, since this procedure requires significant time/manpower.

The fact that laboratory data was collected in parallel with field data will increase the comparability between results observed in the mechanical setup and during free living conditions, respectively.

### Acceleration magnitudes

The aim was to examine acceleration responses in the laboratory under standardized conditions where accelerometer outputs (counts*min^-1^) were comparable to typically values obtained during free-living activities. Compared with validation studies in children [32], the outputs produced in the mechanical setup ranged in locomotion field speed from approximately 4.0 to 8.0 km*h^-1 ^(e.g. the range from walking to running). However, the absence of acceleration responses where only very limited instrument output was produced must be regarded as a limitation in the present study. This is stressed further by findings observed by Brage et al [27] who previously showed that the CSA accelerometer displays larger relative variability at very low accelerations. However, Brage and colleagues suggested that the poor reliability at very low accelerations may be explained by the dead band of the Actigraph (approx. 0.3 m*s^-2^) being different between units, meaning that different units have different lower thresholds at which they begin to register movements. However, acceleration of the human body is expected clearly to exceed that of the dead band, and therefore, in relation to issues linked to calibrated field data the clinical significance of poor reliability at the very low accelerations caused by the dead band is probably very limited. However, different lower threshold of registration might of course affect the number of valid days of measurements since many research groups interpret long bouts of zero activity as non-monitored time. Furthermore, varying lower threshold of registration will potentially have an influence on the amount of time spent in sedentary and/or light intensity categories, depending on how cut points are used.

It might be speculated that numerous periods of zero activity (where children are not moving at all) will attenuate the potential impact of unit-specific calibration when applied to field data, due to the fact that when exposed to no acceleration at all, all instruments will produce the exact same output (i.e., zero). Therefore, the effect of applying unit-specific calibration factors to field data representing the percentage of total registered time spent in high or vigorous activity levels, defined according to Trost et al. [16] was analysed post hoc as the unit-specific calibration factors were applied separately for each epoch being downloaded (data not shown). However, under these circumstances where periods of zero activity are greatly eliminated from field data the exact same result was observed – calibration did not reduce random variation caused by inter-instrumental variability across the examined group of children and adolescents.

### Movement characteristics

The mechanical setup solely offers isolated and standardized sinusoid accelerations. However, children have been reported typically to be involved in many different activities, including different games, jumping, dancing, running, climbing, and biking [33], introducing a wide range of frequencies and accelerations through more complex movements of the human body. These dissimilarities between types of movement, as well as biomechanical differences between subjects, even when involved in the same type of activity, might affect the comparability between inter-instrument variability characterized in the mechanical setup and in field when assessing the complex and heterogeneous behaviour of human locomotion in children and young people. Even when examining reliability using a standardized treadmill protocol, Welk and colleagues [34] found that Actigraph accelerometer counts for a standardized bout of activity can vary by 20% for participants wearing the same monitor and performing the same absolute workload. For example, differences in step frequencies have been reported to explain 11% and 40% of the speed-adjusted variance in Actigraph output in walking and running, respectively [35]. Therefore, the effect of calibration on increased inter-instrumental reliability might be reduced due to an increased between-individual variation caused by differences in step frequencies during free living conditions.

Furthermore, previous Brage et al. [27], found inter-instrument differences to be heteroschedastic in response to the acceleration magnitude, which indicates that inter-instrument variability is related to the frequency and/or magnitude of movement. Similar findings have been reported by Jakicic et al. [36] who found that inter-instrument reliability in the TriTrac-R3D accelerometer appeared to depend on the specific type of PA being assessed.

### Optimal measuring axis of movement

When trying to achieve successful calibration it is important to optimize the parallelism between the measuring axis of the instrument and the axis of movement actually experienced. When calibration and quality checks were performed in the laboratory a standardized attachment of the instruments to the plate at the calibration machine was performed in order to ensure that the registration of acceleration along the vertical axis was optimized. Ideally, every child would wear the accelerometer at the exact same angle in the field. However, even though participants were carefully instructed how to wear the accelerometer, rather individual attachments to the body must be expected, and instrument position might change as a result of lose attachment combined with body movements (which in the end will contribute to an increased random variation in the field). The scope of this problem is illustrated by previous findings showing a reduced accelerometer output of 6%, 16%, and 29% when the optimal angle at the axis of measurement was reduced by 15°, 30°, and 45°, respectively, during standardized conditions in the laboratory [26].

### Agreement between raw and calibrated field data

The amount of variation introduced to field data after applying unit-specific calibration factors was estimated to be only 1.1% and 4.2%, when compared to the total amount of variation in HPA in children and adolescents, respectively. This amount of variation must be considered to be small, especially considering the size of the reproducibility coefficient (R) of a 4-day period previously observed in the children and adolescents examined in the present study. In children, R was found to be approximately 0.65 [31], whereas R was found to be approximately 0.70 in adolescents (unpublished data).

The high correlation between raw and calibrated field data observed in the present study is probably explained by a combination of an improved data quality due to the repeated quality checks and the presence of other major sources of variation (e.g. biological variation, day to day variation, seasonal variation, and poor compliance with correct mounting of the devise to the body), meaning that the inter-instrumental variability will be relatively small when compared to the total amount of variation in field data.

In children who were measured with the MTI instruments, the Bland-Altman plot showed that relative 95% limits of agreement between raw and calibrated instrument output in the field was approximately ± 5.5%. In adolescents measured with the CSA monitors, however, relative limits of agreement showed that 95% of all subjects stayed within a wider range of approximately ± 13% when comparing raw and calibrated field output. A number of outliers caused the limits of agreement in adolescents to be slightly skewed and increased.

Theoretically, ideal calibration factors applied to instruments with zero intra-instrument variation would cause inter-instrumental variability to disappear when examined under standardized conditions in the mechanical setup in the laboratory. However, even though inter-instrument variability was substantially reduced after applying the calibration factors, considerable inter-instrument variations were still observed when examined under standardized conditions in the laboratory. Therefore, even though we would assume that participants whose activity level changed considerable after calibration actually achieved a HPA level closer to their "true" level if monitored with no measurement error at all, the calibration factors estimated and applied in this study will include residual standardized unit-specific test-re-test variation, and could therefore in theory also add to the random variation.

Furthermore, we speculate that unit-specific calibration factors estimated in the laboratory not fully reflect inter-instrument variations in the field. This, in combination with the presence of other major sources of variation, indicates that the Bland-Altman plot only to a certain degree will capture the "true" individual diversity between raw field data and field data obtained without any measurement error. Nevertheless the outliers, which were observed, are probably explained by repeated measurements with one or few units with particular poor reliability. This highlights the importance of performing continuous calibration checks according to an a priori limit of variability.

### Changed inter-instrumental reliability over time

Significantly non-homogeneous standard deviations with increasing size over time were observed in the group of MTI instruments when exposed to standardized accelerations in the mechanical setup. This indicates a modestly reduced inter-instrument reliability throughout the data collection period. Although the SD increased slightly over time in the CSA instruments, a significant heterogeneous pattern could not be observed. It should be noted that when the MTI instruments were calibrated the first time in November no instrument had yet been sent into the field. Therefore, we speculate that the reduced reliability over time partly might be the result of mechanical wear on the cantilevered moving arm (the accelerometer sensor) caused by everyday movements and instrument shocks.

In the group of CSA instruments, inter-instrument variability was found to be rather high to begin with. However, it should be noted that the somewhat older CSA instruments had been used in another study before the first calibration was performed in November 2003. As time went by from November 2003 to March 2004, inter-instrument reliability in the MTI instruments was approaching the level of the CSA instruments.

### Comparing acceleration responses between MTI and CSA instruments

The Actigraph count output has previously been found to increase as frequency decreases at a given acceleration [27,28]. Therefore, the fact that in the present study CSA instruments were exposed to slightly higher frequencies compared to the MTI instruments, potentially challenges the validity of our results indicating a batch effect. However, when identical frequencies and acceleration magnitudes were applied post hoc in June 2004, the MTI instruments displayed a significantly (p < 0.001) increased acceleration response of 9.50%, when compared to the group of CSA instruments. Indications of batch/lot effects have also previously been reported by Esliger et al. [28] who compared mean accelerometer output in six testing conditions in a mechanical setup.

To test whether the different acceleration response observed between the batches of MTI- and CSA instruments in the present study was due to the past use of CSA instruments, the mean acceleration response was compared post hoc in the mechanical setup in 2006 immediately after all CSA units were calibrated according to the spinning procedure recommended by the manufacturer [29]. Results revealed a significantly (p = 0.002) increased mean acceleration response of 10.7% in the MTI instruments, indicating that the diversity previously observed three years earlier apparently mirrored a more universal disparity across the two generations of instruments.

## Conclusion

In conclusion, our results indicate that unit-specific calibration factors, estimated on the basis of acceleration responses in a mechanical setup producing standardized sinusoidal movements, should be considered as rather ineffectual when applied to data collected during free living conditions in children/adolescents. However, the effect of calibration seems to increase slightly over time with increasing instrument age. The inter-instrumental variability was relatively small when compared to the total amount of variation in field data, and in all probability, the effect of calibration was attenuated in the field by other major sources of variation.

Observations from the standardized mechanical setup indicate that increased inter-instrumental variability is to be expected almost instantly when accelerometers are applied during free living conditions in the field. Furthermore, identical acceleration responses can not be expected when comparing different generations, or batches, of the Actigraph accelerometers. Therefore, investigators are advised to be cautious when interpreting and comparing PA data within and between studies where identical instruments have not been used.

For future studies where PA is monitored by the use of accelerometers, we strongly recommend for all research groups that the acceleration response for the specific population of instruments being used is determined before field testing commences. This should prevent biased results due to batch effects. Furthermore, the point is not to discourage instrument evaluation in the laboratory, as we strongly suggest that a simple and time-efficient quality check should be performed in all units each time they are returned from the field. This procedure will prevent broken instruments, defined according to an a priori limit of variability (e.g. mean difference >5%), from being returned to the field again before being repaired.

## Competing interests

The author(s) declare that they have no competing interests.

## Authors' contributions

NCM, LK, and KF conceived of the study, and NCM and LK conceived of the statistical analyses. NCM, PLK, LBA, and NW choose the experimental design and techniques, and KF was responsible for the overall coordination of the study. NCM and PLK did the data processing and performed the data analyses. All authors contributed to the scientific conclusions based on the interpretation of statistical results. NCM and LK wrote the paper, and all authors contributed to critical revision of the paper. All authors approved the final manuscript.

## Pre-publication history

The pre-publication history for this paper can be accessed here:


